# Polyubiquitination of p62/SQSTM1 is a prerequisite for Fas/CD95 aggregation to promote caspase-dependent apoptosis in cadmium-exposed mouse monocyte RAW264.7 cells

**DOI:** 10.1038/s41598-019-48684-2

**Published:** 2019-08-22

**Authors:** Ki-Tae Jung, Seon-Hee Oh

**Affiliations:** 1 Department of Anesthesiology and Pain Medicine, School of Medicine, Chosun University, 309 Pilmundaero, Dong-gu, Gwangju 501-759 Korea; 2 School of Medicine, Chosun University, 309 Pilmundaero, Dong-gu, Gwangju 501-759 Korea

**Keywords:** Apoptosis, Protein aggregation

## Abstract

Cadmium(Cd) induces cytotoxicity via autophagy-induced apoptosis in non-activated mouse monocytes; however, the molecular mechanism remains unclear. Here, we show that autophagy induces Fas (CD95/APO-1)-mediated apoptosis by promoting accumulation of p62/SQSTM1 in response to Cd. Cd produced tumor necrosis factor (TNF)-α, peaking at 6 h, and exhibiting a concentration-dependent increase. Immunoblot analysis revealed polyubiquitinated (polyUb) full-length Fas (antibody clone G-9) and reduced cytosolic Fas (antibody clone M-20) in Cd-exposed RAW264.7 cells. The accumulation of polyUb-Fas was transient and positively correlated with polyUb-p62 and polyUb-proteins. Autophagy inhibition via chemical and genetic modulation suppressed Cd-induced polyUb-p62, polyUb-Fas, and polyUb-protein levels, whereas the level of cytosolic Fas recovered to that of the control. Immunofluorescence (IF) staining for full-length Fas, p62, and ubiquitin revealed an aggregated pattern in Cd-induced apoptotic cells, which was inhibited by blocking autophagy. Fas colocalized with microtubule-associated protein 1 light chain (LC)-3B. IF staining and immunoprecipitation assays revealed colocalization and interaction among p62, Ub, and Fas. Knockdown of p62 reduced the binding of Ub and Fas. Together, these data suggest that polyUb-p62 targets Fas and recruits it to autophagosomes, where Fas transiently aggregates to promote apoptosis and is degraded with polyUb-p62. In conclusion, autophagy regulates C-terminal cytosolic Fas aggregation via p62 polyubiquitination, which is required for apoptosis and may play a critical role in the production of select cytokines.

## Introduction

The cell death receptor, a member of the tumor necrosis factor receptor (TNFR) superfamily, consists of tumor necrosis factor receptor (TNFR)-1, Fas (CD95/APO-1), and the tumor necrosis factor-related apoptosis-inducing ligand death receptors DR4 and DR5^1^. Fas is one of the best characterized death receptors; it triggers downstream signaling pathways by binding with cytoplasmic death domain (DD)-interacting molecules, such as Fas-associated death domain (FADD)^2^, death-associated protein 6 (Daxx)^3^, and RIP^4^. Upon activation, which occurs after binding with its ligand or agonistic anti-Fas antibodies^5^, Fas forms a death-inducing signaling complex (DISC) by binding with caspase-8 and FADD in the plasma membrane, and transduces downstream caspase activation^2^. In the immune system, Fas-mediated apoptosis plays a critical role in lymphocyte maturation and homeostasis^6^. For example, mutation of Fas and its ligand causes lymphoproliferative disease and autoimmune disorders in mice^7,8^. Fas is also implicated in the activation-induced cell death of T lymphocytes and cytotoxic T lymphocytes, and natural killer cell-mediated cytotoxicity against a variety of tumor- and virus-infected cells^9,10^. Therefore, the expression of Fas is important for triggering downstream signaling pathways. Fas-associated protein factor (FAF1) is involved in apoptosis by interacting with the cytoplasmic domain of Fas; thus, its overexpression induces apoptosis^11^. Fas-associated phosphatase (Fap)-1 acts as a negative regulator of apoptosis by binding with a C-terminal negative regulatory domain of Fas^12,13^, which inhibits the surface expression of Fas and increases the intracellular pool^14,15^. Thus, the level of Fap-1 expression may be positively correlated with the resistance of human tumors to Fas-mediated apoptosis^15–17^. In immune cells, Fap-1 has been implicated in cell survival against immune surveillance^18,19^. It has also been reported that autophagy specifically modulates Fas-mediated apoptosis via Fap-1, in which Fap-1 is targeted by p62 for autophagic degradation^20^. Furthermore, the autophagosome-interacting protein microtubule-associated protein 1 light chain-3B (LC3B) regulates apoptosis by interacting with Fas in cigarette smoke-exposed human epithelial Beas-2B cells^21^. However, the mechanism by which Fas protein is modulated to trigger apoptosis in immune cells remains unclear.

Autophagy and apoptosis are programmed cell death pathways that are induced by different molecular mechanisms. Despite marked differences in these pathways, a functional relationship between them has been reported. Both signaling pathways are interconnected by the same regulators and may be activated by simultaneous processes or differential time sequences against a variety of stresses^22^. Anti-apoptotic BCL family proteins, such as BCL-XL and BCL2, inhibit autophagy by binding with Beclin 1/Atg6, which is involved in the formation of the autophagic vesicle^23–25^. Proapoptotic p53 also induces autophagy via increased expression of its target gene, damage-regulated autophagy modulator^26^. FADD plays a role in the formation of DISC, which is involved in the extrinsic apoptotic pathway, as well as in autophagic cell death by interacting with Atg5^27^. The autophagy adaptor molecule p62 shuttles ubiquitinated proteins to autophagosomes or proteasomes^28^. Furthermore, p62 is a molecular linker between autophagy and apoptosis. Autophagy inhibition upregulates p62 that, in turn, promotes caspase-8-dependent apoptosis^29^. Although previous studies have demonstrated molecular connections between autophagy and apoptosis, the relationship between the death receptor and autophagy remains unknown.

Cadmium (Cd), a toxic heavy metal, causes serious health problems. A number of studies have reported the toxic effects of Cd, but the impact on the immune system is uncertain. Since low doses of Cd is more effective in cell proliferation^30,31^, our study focused on cell death by toxic doses of Cd. Cd toxicity is associated with different modes of cell death, including autophagy, apoptosis, and necrosis, which can impair the immune response^32^. We previously found that Cd affects the immune response via autophagy-mediated apoptosis in RAW264.7 cells^33^. Furthermore, Fas plays an important role in quality control of immune cells including macrophage^34^. Therefore, the aim of this study was to clarify the relationship between Fas signaling and autophagy in Cd-induced toxicity. For this purpose, we used the monocytes/macrophage cell line Raw264.7, which plays a central role in the activation of the immune system^35^.

## Results

### Effects of Cd on Fas expression and cytokine production

**Figure 1:**
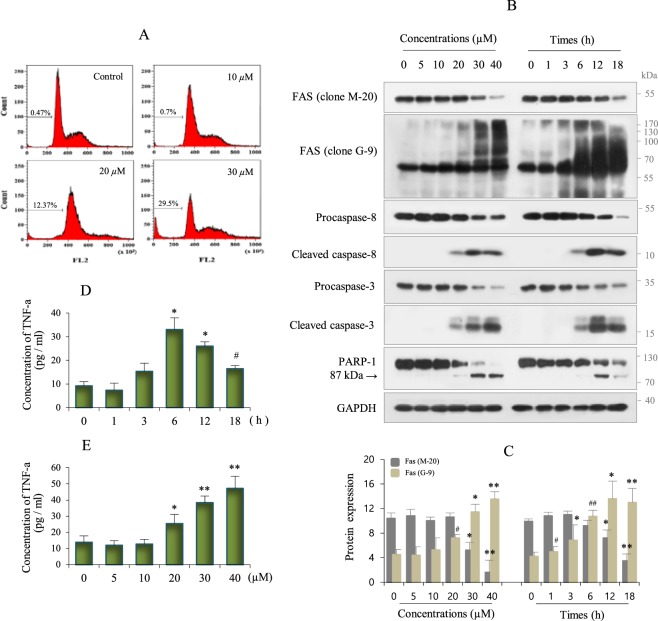
Fas expression and TNF-α production in Cd-exposed Raw264.7 cells. (**A**) Cells were treated with an increasing concentrations of Cd for 18 h, and analyzed for apoptotic sub-G1 by flow cytometry. (**B**) Cells were treated with an increasing concentrations of Cd for 18 h, and with IC_50_ concentrations (30 µM) for up to 18 h and harvested, lysed, and expressions of indicated proteins were assessed by immunoblotting. GAPDH was used as the loading control. Data shown are presentative of over three separate experiments. (**C**) Cells were treated with Cd as described in B, and the expression level of Fas protein (antibody clone M-20 and antibody clone G-9) was quantified by densitometry, and normalized to GAPDH. Data are represented as mean ± SD (n = 3). *P* values were calculated for Cd-exposed cells *versus* control cells. *p < 0.05; **p < 0.005; ^#^*p* < 0.02; ^##^*p* < 0.002 (**D**,**E**) Cells were treated with Cd (30 µM) for up to 18 h, and with different concentrations for 6 h, and then supernatants were collected and analyzed for TNF-a concentrations secreted by ELISA as described in the methods. Data represent the mean ± SD (n = 3). *p < 0.05; **p < 0.005; ^#^*p* < 0.02.

Damage to RAW264.7 cells caused by Cd exposure was examined by fluorescence-activated cell sorting (FACS) analysis. The apoptotic sub-G1 population increased in a Cd-dependent manner (29.5% following treatment with 30 µM Cd) (Fig. 1A). At the molecular level, Cd induced apoptosis in RAW264.7 cells by activation of caspase-8, caspase-3, and poly (ADP-ribose) polymerase (PARP)-1 in a dose- and time-dependent manner (Fig. 1B, Supplememtary Fig. S1). However, western blot analysis revealed a decrease in C-terminal cytosolic Fas (clone M-20) that was dependent on Cd concentration and exposure time used the half maximal inhibitory concentration (IC_50_) of Cd (approximately 30 µM). Whereas, the full-length Fas (antibody clone G-9) was detected as high molecular weight bands, which increased in a dose- and time-dependent manner (Fig. 1B,C, Supplememtary Fig. S2). These results indicate that Cd-induced apoptosis may be caused by a conformational change in C-terminal intercellular Fas.

To elucidate the effect of Cd on the selected inflammatory markers, we assessed the levels of TNF-α and interleukin (IL)-10 in incubation medium at different times and following treatment with various concentrations of Cd by enzyme-linked immunosorbent assay. TNF-α peaked at 6 h (by approximately 3.6-fold and 2-fold, respectively, compared to the control) and then decreased in cells treated with 30 µM Cd (Fig. 1D). The production of TNF-α after incubation for 6 h with different concentrations of Cd showed a concentration-dependent increase (Fig. 1E). However, Cd exposure did not affect IL-10 production (data not shown). These results indicate that Cd affects the immune response via the production of select cytokines.

### Cd exposure results in transient accumulation of polyUb-p62 and p62 monomer via impaired autophagic degradation

**Figure 2:**
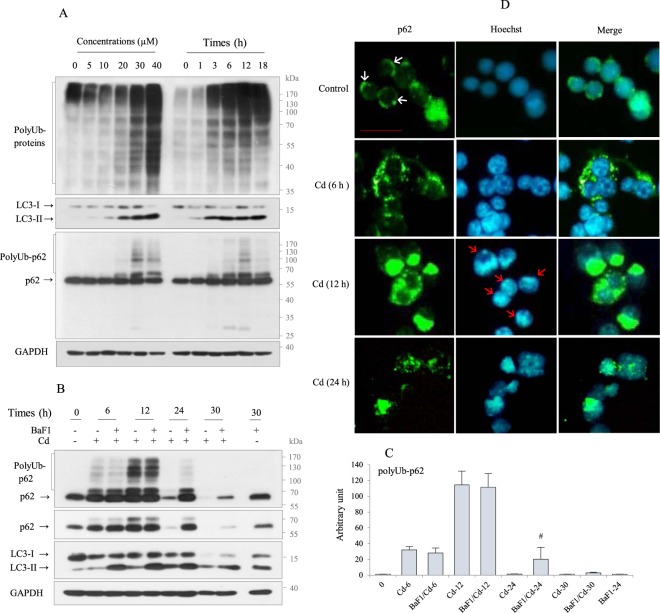
Cd caused p62 polyubiquitination in Raw264.7 cells. (**A**) Cells were treated with an increasing concentrations of Cd for 18 h, and with 30 µM for up to 18 h, harvested, lysed, and expressions of indicated proteins were assessed by immunoblotting. (**B, C**) Cells were exposed Cd (27 µM) with or without pretreatment with BaF1 (10 nM) for 2 h and harvested at different times, and expressions of indicated proteins were assessed by immunoblotting. PolyUb-p62 level was quantified by densitometry and normalized to GAPDH. Data are represented as mean ± SD (n = 3). ^#^*p* < 0.02. (**D**) Cells cultured on coverslips were exposed with Cd (30 µM) for 6, 12, and 24 h, respectively, prior to fixation and stained with p62 antibody (green) and FITC as the secondary antibodies. Nuclei were counterstained with Hoechst 33342 (blue) and acquired by fluorescence microscope (Nikon, Eclipse TE300). White arrows indicate p62 localized in the perinuclear area. Red arrows indicate apoptotic nuclei. Cells were imaged using a 40X objective. Scale, 25 µm. GAPDH was used as the loading control. Data shown are presentative of three separate experiments.

p62 plays an important role in transporting polyUb-proteins to the proteasome and autophagosomes for degradation^28^. We thus examined the mechanism by which p62 regulates the stability of Fas protein during Cd exposure. First, the levels of polyUb-proteins were analyzed by Western blotting. In the concentration-dependent experiment, the levels of polyUb-proteins markedly increased in cells exposed to >20 µM Cd. The time-course experiment used 30 µM Cd (IC_50_) and revealed that polyUb-proteins began to accumulate at 1 h of Cd exposure and peaked at 12 h. Cd exposure also induced autophagy in RAW264.7 cells in a dose- and time-dependent manner, as determined by conversion from LC3-I to LC3-II, which was induced as early as 1 h following Cd exposure and sustained up to 18 h, indicating that autophagy is an earlier event than apoptosis. During autophagy, p62, an autophagy adaptor molecule, is degraded together with its targets within autophagolysosomes. However, p62 accumulated as itself as well as its polyUb-conjugated form and did not observe a noticeable amount of bands below 55 kDa in Cd-exposed RAW264.7 cells (Fig. 2A, Supplememtary Fig. S3). The amount of polyUb-p62 positively correlated with total polyUb-protein levels (Fig. 2A, Supplememtary Fig. S4), implying that autophagic degradation were blocked. To determine the fate of the p62 monomer and polyUb-p62 during Cd exposure, cells were exposed to Cd for up to 30 h. The p62 monomer began to decrease after 12 h of Cd exposure, which was accompanied by a decrease in polyUb-p62 and polyUb-proteins, and the LC3-II level was sustained for up to 30 h, indicating that autophagic degradation begins after 12 h of Cd exposure (Supplementary Fig. S5). These results were further substantiated by blocking autophagic flux through 30 h using BaF1, a chemical autophagy inhibitor, which causes LC3-II accumulation. Pretreatment with BaF1 before adding Cd (27 µM, a lower concentration than IC_50_) for 6 h and 12 h, respectively, did not affect Cd-induced p62 monomer and polyUb-p62 levels compared to Cd-exposed cells for each. However, Cd exposure for 24 h, and 30 h led to a marked decrease of p62 and polyUb-p62, which were partially, not completely, recovered by BaF1 pretreatment (Fig. 2B, C), indicating that accumulation of polyUb-p62 and p62 monomer within 12 h of Cd exposure caused by impaired autophagic degradation. Consistently, we found that proteasome inhibition via MG132 (2–8 µM) did not affect the level of Cd-induced polyUb-p62 (Supplementary Fig. S6). These results imply that p62-targeted proteins during Cd exposure transferred to autophagosomes but not to the proteasome. Indeed, we found that interaction between p62 and LC3 by IP analysis (Supplementary Fig. S7A, B).

Next, we examined p62 expression by immunofluorescence (IF). In untreated control cells, p62 mainly localized in the cytoplasm as a ring pattern surrounding the nucleus (white arrows) and showed a faintly diffused staining pattern in the nucleus. By contrast, at 6 h of Cd exposure, p62 accumulation was observed as a granular pattern in the cytosol. However, after 12 h of Cd exposure, many cells showed apoptotic nuclear changes (red arrows), such as chromatin condensation and nuclear cleavage, and p62 exhibited a strong and aggregated immunofluorescence staining pattern. Most cells treated with Cd for 24 h showed an apoptotic nuclear change and markedly reduced p62 staining (Fig. 2D). These results suggest a specific role for p62 in the stability of target proteins.

### p62 interacts with polyUb in response to Cd

**Figure 3:**
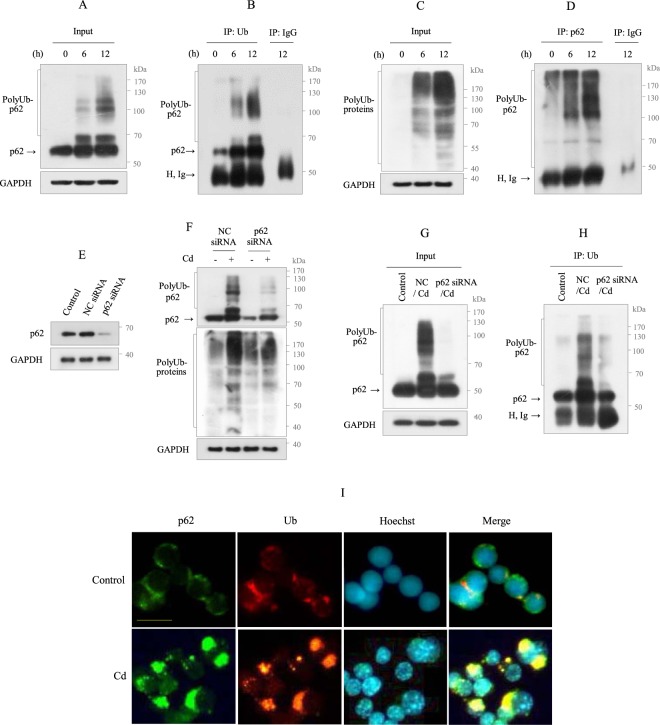
p62 binds with polyUb in Cd-exposed Raw264.7 cells. (**A, B**) Cells were treated with Cd (30 µM) for 6 and 12 h, respectively. Lysates were immunoblotted for p62 and the other aliquots (600 µg) were immunoprecipitated with Ub antibody and mouse IgG, and immunoblotted for p62. (**C, D**) Cells were treated as described in above, and the lysates were immunoblotted for Ub and the other aliquot (600 µg) were immuniprecipitated with p62 antibody and mouse IgG, and immunoblotted for Ub. (**E**) After transfection with negative control (NC) siRNA and *p62*-specific siRNA, cells were cultured for 24 h, and transfection efficiency was evaluated by immunoblotting for p62 protein. (**F**) Cells transfected with NC siRNA and p62 siRNA were exposed to Cd (30 µM) for 12 h, and immunoblotted for the indicated proteins. (**G, H**) Cells were transfected as described in above prior to Cd exposure 12 h and the lysates were immunoblotted for p62 and the other aliquot (600 µg) was immuniprecipitated with Ub antibody and immunoblotted for p62. All data are representative of over three independent experiments. H, Ig indicates immunoglobulin heavy chain. (**I**) Cells cultured on coverslips were exposed with Cd for 12 h prior to fixation and stained with p62 antibody (green) and Ub antibody (red), and FITC and rhodamin as the secondary antibody, respectively. Nuclei were counterstained with Hoechst 33342 (blue) and photographed. Cells were imaged using a 40X objective. The scale bar is 25 µm.

p62 is a multifunctional protein that contains an LC3-interacting region (LIR), a ubiquitin-binding domain, and a proteasome interacting domain (Phox and Bem 1p, PB1)^36^. Thus, to determine whether p62 accumulation is associated with ubiquitination in Cd-exposed RAW264.7 cells, immunoprecipitations (IP) for Ub and mouse IgG were performed, followed by immunoblotting with p62 and vice versa. The results revealed an interaction between ubiquitin and p62 (Fig. 3A–D), which was confirmed in p62-knockdown cells. Transfection with a specific small interfering RNA (siRNA) for p62 markedly suppressed p62 (Fig. 3E). Cd exposure to p62-knockdown cells resulted in decreased levels of polyUb-p62 and total polyUb-proteins compared to Cd-exposed control siRNA-transfected cells (Fig. 3F). Moreover, the IP assay for Ub with p62-knockdown cell lysates revealed a reduced binding level of polyUb-p62 (Fig. 3G, H). The interaction between p62 and Ub was further confirmed by IF staining. Both proteins appeared in the cytoplasm, exhibiting a ring pattern surrounding the nucleus (p62, green; Ub, red). However, in Cd-exposed cells, a strong and aggregated staining pattern for both proteins, which were co-localized in the cytosol, was observed (Fig. 3I). Together, these results suggest that p62 binds polyUb and may play a critical role in Cd-induced cell death.

### Autophagy regulates p62 polyubiquitination and the Fas signaling pathway

**Figure 4:**
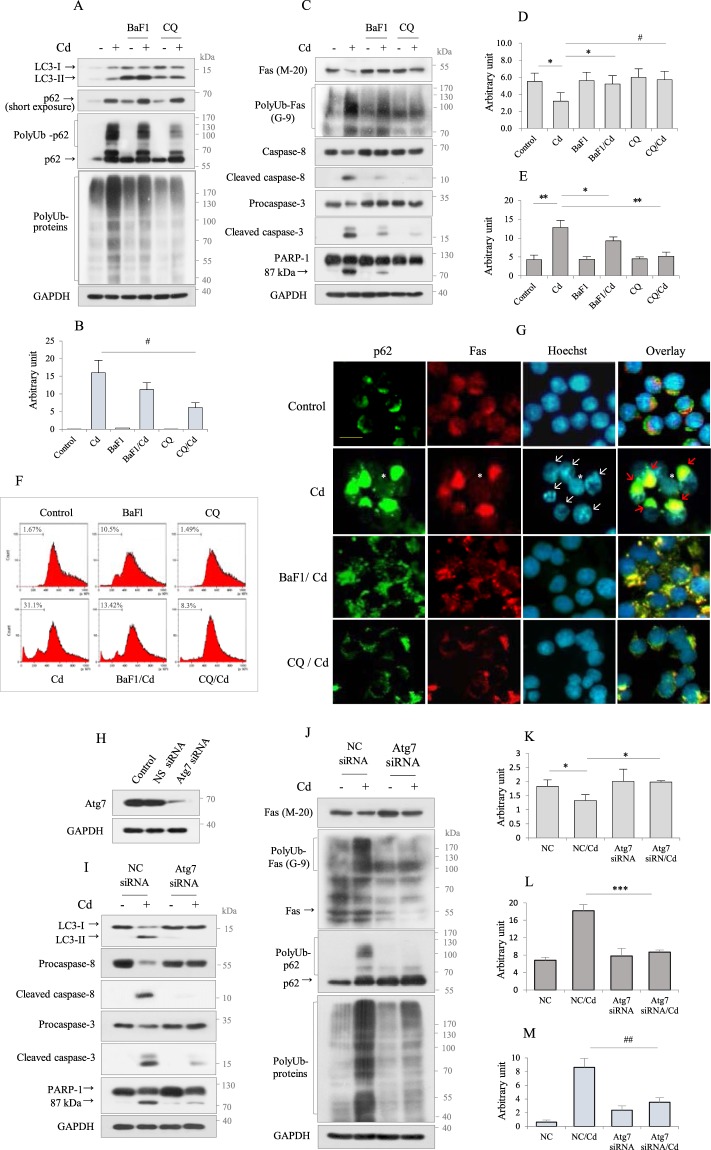
Autophagy regulates polyubiquitination of p62 and Fas in Cd-exposed Raw264.7cells. (**A, C**) Cells were pretreated with autophagy inhibitors BaF1 (10 nM) and CQ (100 µM) for 2 h and followed by Cd (30 µM) treatment for 12 h, and immunoblotted for indicated proteins. GAPDH was used as the loading control. (**B, D, E)** The levels for polyUb-p62 (**B**), Fas protein (antibody clone M-20) (**D**), and PolyUb-Fas (antibody clone G-9) (**E**) were quantified by densitometry and normalized to GAPDH in arbitrary units. The results are given as the mean ± SD (n = 3). *p < 0.05; **p < 0.005; ^#^*p* < 0.02. (**F**) Cells were treated as described in above, and analyzed for apoptotic sub-G1 by flow cytometry. Representative data from three different experiments are shown. (**G**) Cells cultured on coverslips were exposed with Cd for 12 h prior to fixation and stained with p62 antibody (green) and Fas antibody (red), and FITC and rhodamin as the secondary antibody, respectively. Nuclei were counterstained with Hoechst 33342 (blue). White and red arrows indicate apoptotic nuclei and aggregated staining for p62 and Fas, respectively. Asterisk indicates normal cells. Cells were imaged using a 40X objective. The scale bar is 25 µm. (**H**) After transfection with negative control (NC) siRNA and *atg7*-specific siRNA, transfection efficiency of Atg7 was evaluated by Western blot analysis at 24 h of transfection. (**I, J**) Cells transfected with NC siRNA and *atg7*-specific siRNA were exposed to Cd (30 µM) for 12 h, and harvested, lysed, and amount of indicated proteins were assessed by immunoblotting. GAPDH was used as the loading control. Quantitative analyses for protein level of Fas (M-20)(K), PolyUb-Fas (G-9)(L), and polyUb-p62 (M) were performed by densitometry and normalized to GAPDH. Data represent the mean ± SD (n = 3). *p < 0.05; ***p < 0.0005; ^##^*p* < 0.002.

Accumulating studies have indicated that autophagy is involved in either cell protection or cell death depending on the stress conditions. We previously showed that Cd-induced autophagy promoted apoptosis in RAW264.7 cells^33^. To investigate the role of autophagy in Cd-induced Fas signaling, autophagy was inhibited with chemical inhibitors, including BaF1 or chloroquine (CQ) before adding Cd for 12 h, when there is a peak in polyUb-p62 level. Both inhibitors caused a further accumulation of Cd-induced LC3-II, with a slight accumulation of p62 monomer (short exposure) compared to Cd-exposed cells. However, both inhibitors reduced the levels of polyUb-p62 and polyUb-proteins, and the effects of CQ were much greater (Fig. 4A, B). Interestingly, Western blot and densitometric analyses revealed that autophagy inhibition recovered the level of Cd-induced downregulated cytosolic Fas (M-20) to that of the control, reduced the amount of polyUb-Fas (G-9), and partially inhibited apoptosis by suppressing caspase-8, caspase-3, and PARP-1 activation (Fig. 4C–E), which was further confirmed by FACS analysis. The Cd-induced sub-G1 population (31.1%) decreased following treatment with BaF1 and CQ (13.42% and 8.30%, respectively) (Fig. 4F). The involvement of autophagy in the regulation of Fas and polyUb-p62 was examined by immunocytochemistry. In non-treated control cells, p62 (green) and Fas (red) exhibited a ring-staining pattern surrounding the nucleus. However, in cells with an apoptotic nuclear appearance following Cd exposure (white arrows), both proteins exhibited a strong and aggregated staining pattern and colocalized in the cytosol (red arrows). However, non-apoptotic cells did not exhibit p62 and Fas accumulation (white asterisk) (Fig. 4G). Although autophagy inhibition by BaF1 did not cause a marked difference in polyUb-p62 level compared to Cd-exposed cells, the staining pattern was different; p62 appeared in an granular staining pattern along the periphery of the nuclear membrane, implying that autophagy might play a role in aggregation of polyUb-p62. The effect of autophagy on poly-ubiquitination of p62 and Fas was further substantiated by inhibiting autophagosome formation via genetic knockdown of the autophagy-related gene, *atg7*. Transfection with siRNA targeting *atg7* markedly reduced the Atg7 protein level (Fig. 4H), suppressed the conversion of LC3-I to LC3-II, and inhibited caspase-8, caspase-3, and PARP-1 cleavage (Fig. 4I, Supplememtary Fig. S8). Consistent with the pharmacological inhibition of autophagy, Atg7 knockdown recovered the level of cytosolic Fas to that of the control and markedly reduced the amount of polyUb-Fas, polyUb-62, and polyUb-proteins, but further accumulated p62 monomer compared to Cd-exposed cells (Fig. 4J–M). These results indicate that p62 polyubiquitination and Fas protein level may be regulated by autophagy.

### Fas is recruited to the autophagosome via targeting by polyUb-p62

**Figure 5:**
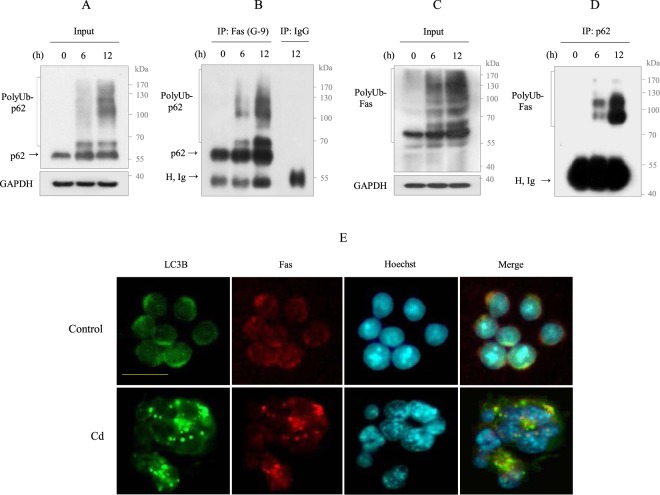
Fas interacts with p62 and colocalized with LC3B in Cd-exposed Raw264.7 cells. (**A, B**) Cells were treated with 30 µM Cd for 6 and 12 h, respectively. Lysates were immunoblotted for p62, and the other aliquot (600 µg) were immuniprecipitated with Fas antibody (antibody clone G-9) and mouse IgG, followed by immunoblotting for p62. (**C, D**) Cells were treated as described in above. Lysates were immunoblotted for Fas (clone G-9), and the other aliquot (600 µg) were immuniprecipitated with p62 antibody and mouse IgG, followed by immunoblotting for Fas (clone G-9). All data shown are presentative of over three separate experiments. H, Ig indicates immunoglobulin heavy chain. GAPDH was used as the loading control. (**E**) Cells cultured on coverslips were exposed with Cd (30 µM) for 12 h prior to fixation and stained with LC3B (green) and Fas (red) antibodies, and FITC and rhodamin as the secondary antibody, respectively. Nuclei were counterstained with Hoechst 33342 (blue) and photographed. Cells were imaged using a 40X objective. The scale bar is 25 µm.

The above results suggest that the downregulation of cytosolic Fas may be associated with Fas aggregation. In this context, p62 can interact with Fas via its N-terminal PBI domain or via C-terminal UBA domain-mediated polyUb. Prolonged exposure of Cd (up to 30 h) resulted in a positive correlation between polyUb-p62 and polyUb-Fas levels (Supplementary Fig. S5). Furthermore, as shown in the IF images in Fig. 4E, the colocalization of p62 and Fas was in the cytosol, suggesting that Fas interacts with p62. To test this interaction, we performed an IP assay for Fas followed by western blot analysis for p62. The IP assay with Cd-exposed cell lysates revealed that Fas interacts with polyUb-p62 and p62 monomer. Interestingly, the interaction between Fas and p62 monomer was also observed in control cells (Fig. 5A, B). This interaction was further confirmed with an IP assay for p62 followed by Western blot analysis for Fas. As expected, an interaction between Fas and polyUb-p62 was observed (Fig. 5C, D). Together, these results indicate that Fas interacts with p62 via p62 UBA domain-binding polyUb. Because p62 interacts directly with LC3B on the phagophore via its LIR domain, we next examined whether the polyUb-p62-Fas complex is incorporated into the autophagosome. The intracellular localization of Fas (red) and LC3B (green) was examined by IF staining. In non-treated control cells, LC3B and Fas were colocalized. However, LC3B and Fas appeared as large dots or exhibited an aggregated staining pattern following Cd exposure and revealed overlapping (Fig. 5E). These results indicate that p62-targeted Fas may be recruited to the autophagosome, which could lead to the aggregation of C-terminal cytosolic Fas.

### PolyUb-p62-mediated Fas aggregation promotes apoptosis

**Figure 6:**
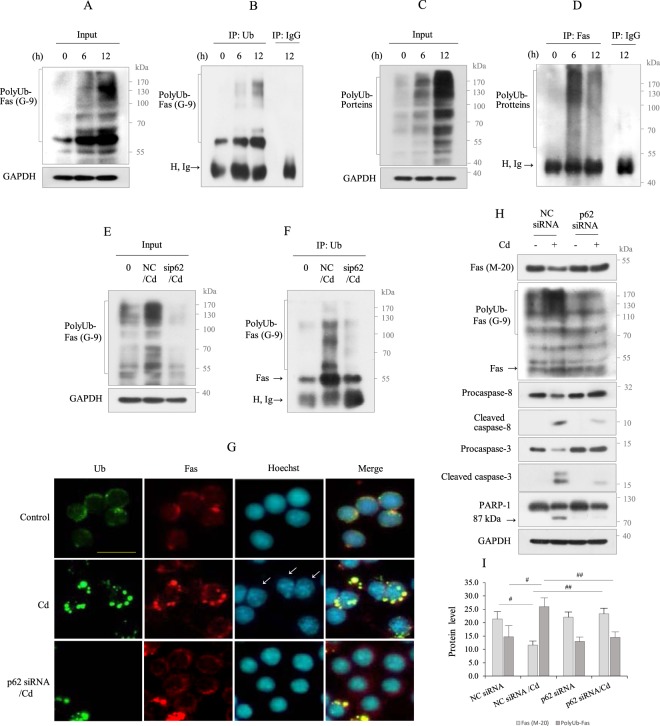
Fas interacts with polyUb, which was regulated by p62. (**A**, **B)** Cells were treated with Cd (30 µM) for 6 and 12 h, respectively. Lysates were immunoblotted for Fas, and the other aliquots (600 µg) were immunoprecipitated with Ub antibody and mouse IgG, followed by immunoblotting for Fas (G-9). (**C, D**) Lysates obtained from above were immunoblotted for Ub, and the other aliquots (600 µg) were immuniprecipitated with Fas (G-9) and mouse IgG, followed by immunoblotting for Ub. GAPDH was used as the loading control. H, Ig indicates immunoglobulin heavy chain. (**E, F**) Cells transfected with negative control (NC) siRNA and *p62*-specific siRNA were exposed to Cd (30 µM) for 12 h, and the lysates were immunoblotted for Fas (G-9), and the other aliquot (600 µg) were immuniprecipitated with Ub antibody, followed by immunoblotting for Fas (G-9). (**G**) Cells cultured on coverslips were exposed with Cd (30 µM) for 12 h prior to fixation and stained with Ub (green) and Fas antibodies (red), and FITC and rhodamin as the secondary antibody, respectively. Nuclei were counterstained with Hoechst 33342 (blue) and photographed. Arrows indicate nuclei showing apoptotic changes. Cells were imaged using a 40X objective. The scale bar is 25 µm. (**H, I**) Cells transfected with negative control (NC) siRNA and *p62*-specific siRNA were exposed to Cd (30 µM) for 12 h, and the lysates were analyzed for indicated proteins. GAPDH was used as the loading control. All data are representative of over three independent experiments. Quantitative analyses for protein level of Fas (M-20) and PolyUb-Fas (G-9) were performed by densitometry. Data represent the mean ± SD (n = 3). ^#^*p* < 0.02; ^##^*p* < 0.01.

The above results (Figs 3–6) indicate that p62 binds Ub and Fas. Next, we explored whether Fas binds Ub. Because colocalization does not indicate a direct interaction, we investigated whether Fas directly binds Ub by performing an IP assay for Ub and mouse IgG followed by western blot analysis for Fas. As shown in Fig. 6A,B, Fas interacted with Ub. When the IP assay with Fas was analyzed for Ub by western blotting, polyUb was detected (Fig. 6C, D), indicating that Fas interacts directly with Ub.

To explore the functional role of p62 in Fas poly-ubiquitination, p62-knockdown RAW264.7 cells were exposed to Cd, and the lysates were subjected to an IP assay for Ub followed by western blot analysis for Fas. The effect of p62-knockdown was validated, as shown in Fig. 3E. Knockdown of p62 inhibited the interaction between Fas and Ub, indicating that poly-ubiquitination of Fas may be regulated by p62 (Fig. 6E, F). These results were further confirmed by IF staining for Ub and Fas in p62-knockdown cells. In non-treated control cells, Ub (green) and Fas (red) exhibited a ring pattern surrounding the nucleus. In cells exhibiting an apoptotic nuclear feature following Cd exposure (white arrows), Ub and Fas exhibited a strong and aggregated dot pattern, and both proteins completely overlapped, indicating that Fas colocalizes with Ub. In Cd-exposed p62-knockdown cells, the green dots representing p62 nearly disappeared. Although the large, red dots representing Fas observed in Cd-exposed cells disappeared in Cd-exposed p62-knockdown cells, a red ring-staining pattern surrounding the nucleus was observed, which exhibited a stronger intensity than control cells (Fig. 6G). Furthermore, at the biochemical level, p62-knockdown recovered the level of cytoplasmic Fas to that of the control but suppressed polyUb-Fas and subsequently mitigated caspase-8-dependent apoptosis (Fig. 6H). Together, these results indicate that C-terminal cytoplasmic Fas aggregation by Cd exposure may be regulated by p62 polyubiquitination, which may be involved in the apoptotic process.

## Discussion

Previous studies have revealed that Fas receptor aggregation plays a distinct role in triggering the downstream signaling pathway following Fas activation^37–39^. However, the mechanism underlying Fas receptor aggregation remains unclear. Our previous study demonstrated that autophagy plays a critical role in Cd-induced cytotoxicity by upregulating caspase-dependent apoptosis in RAW264.7 cell^33^. Monocytes play a key role in the immune response, which includes the phagocytosis of pathogens, antigen presentation, and the production of cytokines and chemokines^40^, indicating that monocyte cytotoxicity may result in an impaired immune response. For this reason, we used non-activated RAW264.7 cells to investigate the molecular mechanism involved in the immunomodulatory effects of Cd. The present study strongly suggests that Cd-induced apoptosis is regulated by polyUb-p62-mediated Fas aggregation that is promoted by autophagy in Cd-exposed RAW264.7 cells.

Accumulating *in vivo* and *in vitro* studies involving humans and rodents have demonstrated that Cd exerts pro- and anti-inflammatory properties^41^. One major cause for the contradictory findings can be ascribed to the different experimental setups, including the concentrations of Cd used, cell type, and experimental conditions. Nevertheless, Cd cytotoxicity appears to be associated with inflammation. Inflammation is a protective response against cellular injury by various toxic insults or infection through preventing tissue damage and triggering repair, and thus restores the physiological functions of the organs affected by inflammation^42^. Cd exposure affects various cytokines and chemokines, including TNF-α, IL-6, IL-10, IL-1β, IL-1a, and IL-8^41^. In our experiments, TNF-α production peaked at 6 h of Cd exposure (immediately before the intracellular biochemical changes by Cd reached their peak), followed by massive cell death. These results imply that cells respond to Cd toxicity via production of pro-inflammatory cytokine.

As noted, despite the importance of Fas receptor aggregation in the Fas signaling pathway^37–39^, the underlying molecular mechanism of Fas aggregation remains unclear. Extensive cell death by DNA damaging agents, such as cisplatin and γ-irradiation, in Jurkat cells caused by Fas aggregation did not affect FasL expression^37^. Gajate *et al*.^38^ reported a critical role for the Fas cytoplasmic domain, which recruits FADD, procaspase-8, JNK, and Bid into lift rafts in response to the antitumor drug Edelfosine and triggers mitochondrial-mediated apoptosis. Additionally, Wright *et al*.^39^ reported that the cytoplasmic Fas domain interacts with ubiquitin-conjugating enzyme (UBC-FAP), suggesting that UBC-FAP plays a critical role in Fas signal transduction via the ubiquitination of Fas itself or Fas-associated molecules, and further ubiquitination of Fas causes Fas aggregation. In the present study, when Fas was detected by immunoblot analysis using an antibody for clone M-20 raised against C-terminal mouse Fas as an antigenic epitope, its level decreased in a dose- and time-dependent manner. On the other hand, full-length Fas (clone G-9) appeared as a cumulative high molecular weight multi-band. In addition, IP analysis of Fas and ubiquitin confirmed that the band detected by the G-9 antibody could be polyUb-Fas. Also, previous studies that the cytoplasmic domain of Fas interacts with UBC-FAP supported our results^39,43^. Thus, our results suggest that Fas is recruited to proteasomes or autophagosomes via polyubiquitination. However, proteasome inhibition by MG132 (2–8 μM) did not affect the level of Cd-induced polyUb-Fas (Supplementary Fig. S6), which indicates that Fas levels may be dependent on autophagy rather than proteasome degradation. Indeed, autophagy inhibition by chemical inhibitors or genetic modulation before Cd exposure resulted in upregulation of the cytoplasmic Fas level to the basal level, as demonstrated by the clone M-20 antibody, and suppressed polyUb-Fas, as demonstrated by the clone G-9 Fas antibody. A strong and aggregated staining pattern for clone G-9 Fas antibody disappeared following autophagy inhibition. Furthermore, Fas colocalized with LC3B. These results indicate that Fas aggregation is regulated by autophagy. Therefore, the Western blot data for the clone M-20 Fas antibody suggest that during early autophagy, the cytoplasmic domain of Fas may form an aggregate that is stable in sodium dodecyl sulfate and mercaptoethanol, and may cause the accessibility of the M-20 Fas antibody to deteriorate. This time we show that full-length Fas interacts with polyUb by IP analysis, but it is not completely clarified whether the high molecular weight bands detected by the G-9 antibody are polyUb-Fas, so further studies are needed. In addition, we are unable to demonstrate how aggregated cytoplasmic Fas activates caspase-8 and the structural change in aggregated Fas. Thus, these will be important issues to address in our future work. Nevertheless, we confirmed an interaction between Fas and caspase-8 (Supplementary Fig. S9).

Next, we investigated how Fas expression is regulated by autophagy. Similar to the ubiquitin proteasome system, substrate ubiquitination serves as a signal for cargo recognition in selective autophagy. p62 acts as a cargo adaptor for both proteasomal- and autophagy-mediated degradation^28,44^ and targets ubiquitinated substrates via its C-terminal UBA domain^45,46^, indicating that p62 plays a dual role in intracellular protein degradation processes, including ubiquitin-proteasomal and lysosome-dependent autophagy^28^. An ubiquitinated cargo adaptor shuttles ubiquitinated substrates for proteasomal degradation but remains stable itself; thus, if p62-targeted polyUb-substrates are recruited to the proteasome, p62 and Ub should accumulate. In the present study, although proteasome inhibition by MG132 (≥8 *µ*M) led to a slight accumulation of polyUb-proteins, it did not affect the level of p62, polyUb-p62, or polyUb-total Fas in Cd-exposed RAW264.7 cells (Supplementary Fig. S6). Therefore, our data indicate that p62-targeted polyUb-Fas is not recruited to the proteasome.

The N-terminal PB1 protein-protein interaction domain of p62 binds with various proteins containing the PB1 domain and the proteasome, and is involved in self-oligomerization (i.e., binds with ubiquitinated cargo through its UBA domain for autophagosomal degradation)^44,47,48^. In the present study, p62 interacted with Ub, as demonstrated by IP and IF staining, which resulted in their aggregation. Because p62 directly interacts with LC3B, the p62-cargo complex can be incorporated into the autophagosome and subsequently degraded with cargo contents in the lysosomes^49^. Autophagic conversion of LC3B began as early as 1 h of Cd exposure and was sustained for up to 30 h, but p62 monomer began to decrease after 12 h, implying that early autophagy may be implicated in polyUb-p62 accumulation rather than autophagic degradation. Indeed, inhibition of autophagy flux by BaF1 within 12 h of Cd exposure did not affect p62, polyUb-p62, or polyUb-proteins induced by Cd treatment, but BaF1 treatment led to partial accumulation of the three proteins after 12 h of Cd exposure, indicating that autophagic degradation begins after 12 h of Cd exposure. At late times, partial accumulation of the three kinds of proteins might be caused by the incapacity of the inhibitor due to its biological half life or massive degradation of polyUb-p62. Additionally, BaF1 treatment suppressed caspase activation, implying that massive autophagic degradation links to apoptosis. Thus, early autophagy inhibition (i.e., before the decrease of p62 begins) can suppress Fas aggregation or at least delay it. Consistently, inhibition of autophagosome formation via *atg*7 knockdown almost completely inhibit Fas-mediated caspase activation. Additionally, IF staining for p62 and Fas in autophagy-blocked cells markedly reduced the aggregated pattern and inhibited Cd-induced caspase activation. These results support our hypothesis that autophagy plays a dual function in the accumulation and degradation of p62 and its target molecules. Although our study did not elucidate how aggregated Fas in the autophagosome triggers downstream signaling pathways, several of the experiments clearly show that the Fas receptor forms aggregates via autophagy-mediated p62 polyubiquitination.

In conclusion, autophagy-induced apoptosis in RAW264.7 cells plays a critical role in immunomodulation in response to Cd. Early in the process, autophagy leads to polyUb-p62 accumulation to promote Fas aggregation and concomitantly produces proinflammatory cytokines. Therefore, the polyubiquitination of p62 is a prerequisite for Cd-induced Fas-mediated apoptosis, indicating that Cd-induced immunotoxicity may be effectively inhibited by blocking autophagy at an early step.

## Materials and Methods

### Cell culture

RAW264.7 cells, a mouse monocyte/macrophage cell line, were obtained from the American Type Culture Collection (ATCC, no. TIB-71^TM^). The cells were cultured in Dulbecco’s modified Eagle’s medium (DMEM; Invitrogen, MA, USA) supplemented with 10% fetal bovine serum (Invitrogen) supplemented with penicillin and streptomycin. The cells were grown in a humidified incubator with 5% CO_2_ at 37 °C. In all experiments, cells between third and 10th passage were used.

### Reagents and antibodies

Cadmium acetate, bafilomycin A1, chloroquine, propidium iodide (PI), MG132, hoechst 33342 were obtained from Sigma-Aldrich (St. Louis, MO, USA). Antibodies for Clone M-20 Fas (sc-716), clone G-9 Fas (sc-74540), procaspase-3 (sc-7148), ubiquitin (sc-8017), GAPDH (sc-32233), FITC-goat anti-mouse (sc-2010) and rhodamin-goat anti-rabbit (sc-2091) were purchased from Santa Cruz Biotechnology (Santa Cruz, CA, USA). Anti-LC3B (2772), PARP-1 (9532), cleaved caspase-3 (9661), and caspase-8 (4790) purchased from Cell Signaling Technology (Beverly, MA, USA). Antibodies for SQSTM1/p62 (H00008878-M01) and Atg7 (EPR6251) were obtained from Abnova (Taipei, Taiwan) and Epitamics (Burlingame, CA, USA), respectively. Unnoted chemicals were obtained from Sigma.

### Flow cytometric analysis

Cells were harvested and washed twice with cold phosphate-buffered saline (PBS). To analyze the percentage of nuclei with hypodiploid content (Sub-G1), nuclei were stained by using PI and at least 20,000 events were analyzed by a FACScan and Kaluza Analysis 1.5a (Beckman Coulter).

### Cytokine detection

Concentration of TNF-α was measured by using specific ELISA kit (Invitrogen, Carlsbad, CA). Cells were plated at a density of 1.5 × 10^6^ cells/mL in 6-well plate with 1 mL of culture medium. After treatment with chemicals, supernatants were collected and analyzed for TNF-α as described in the manufacturer’s instructions. Finally, optical density was quantified using a conventional microplate reader and the concentrations were calculated in pg/mL by applying the Magellan Software 5 (Tecan Safire2, M¨annedorf, Switzerland).

### RNA interference

The target-specific siRNAs (small interfering RNA) and negative control siRNA were transfected to Raw264.7 cells using Lipofectamine^TM^ RNAiMAX reagent (Invitrogen) as described in the manufacturer’s instructions. Target siRNAs for Atg7 and p62 used in experiments were as follows: mouse Atg7 (5′-GGAGUCACAGCUCUUCCUUTT-3′) and mouse p62 (5′-CTTGTAGTTGCATCACGTA-3′).

### Immunoblot analysis and immunoprecipitation (IP)

After treatment, cells were harvested, lyzed, and processed for Western blot analysis and immunoprecipitation as previously described^50^. For immunoblot analysis, 15–30 μg of proteins were resolved by 10–12% SDS-PAGE and transferred to PVDF membrane (Millipore). The membranes were probed with the respective primary antibodies and secondary antibodies, and the images were detected using chemiluminescent substrate (Millipore). For IP analysis, cells were lysed in IP lysis buffer (50 mM Tris-HCl [pH 7.4], 250 mM NaCl, 0.25% Triton X-100, 10% glycerol) containing protease inhibitor cocktail (Roche). Proteins (600 μg) were precleared with 50% protein G Plus-agarose bead (Santa Cruz Biotechnology) for 10 min and centrifuged at 12,000 × g at 4 °C for 10 min. The supernatants were incubated with the indicated primary antibodies for overnight at 4 °C on a shaker. IP-antibody complexes were then captured on protein G Plus-agarose bead, and analyzed by SDS-PAGE. Semi-quantification of protein detection was analyzed by Image J program (National Institutes of Health, Bethesda, USA). The relative amount of each protein was normalized to the ratio between the value for each protein and the value for the respective loading control.

### Immunofluorescence (IF) staining

IF staining was performed as described previously^50^. Briefly, cells cultured on the cover slip were fixed in 10% neutral-buffered formalin for 10 min on ice and washed with PBS, permeabilized with 0.05% Triton X-100 for 10 min, washed with PBS, and blocked with 2% bovine serum albumin. Cells were incubated with primary antibodies and fluorescent secondary antibodies. Nucleus was stained with hoechst 33342 (1 μg/ml) and mounted using Vectashield^®^ (Vector Labs, Burlingame), and images were captured using fluorescence microscope (Nicon Eclips TE300).

### Statistical analysis

All experiments were repeated at least three times, and values are expressed as the mean ± standard deviation (SD). The statistical significance of the difference between experimental groups was determined by one-way ANOVA with a post-hoc test. A value of *p* < 0.05 was considered statistically significant.

## Supplementary information

Supplementary figure
