# Supplementary material for: Polyubiquitination of p62/SQSTM1 is a prerequisite for Fas/CD95 aggregation to promote caspase-dependent apoptosis in cadmium-exposed mouse monocyte RAW264.7 cells
Source: Sci Rep. 2019 Aug 22;9:12240. doi: 10.1038/s41598-019-48684-2 (PMC6706394; doi:10.1038/s41598-019-48684-2)
Supplement: Supplementary file 1 — Supplementary figure [file 41598_2019_48684_MOESM1_ESM.pdf]

Polyubiquitination of p62/SQSTM1 is a prerequisite for Fas/CD95 aggregation to promote caspase-dependent apoptosis in cadmium-exposed mouse monocyte RAW264.7 cells

Ki-Tae Jung[‡] and Seon-Hee Oh[£,*]

‡Department of Anesthesiology and Pain Medicine, School of Medicine, Chosun University, 309 Pilmundaero, Dong-gu, Gwangju 501-759, Korea

[£] School of Medicine, Chosun University, 309 Pilmundaero, Dong-gu, Gwangju 501-759, Korea

Supplementary Fig. S1 (Oh)

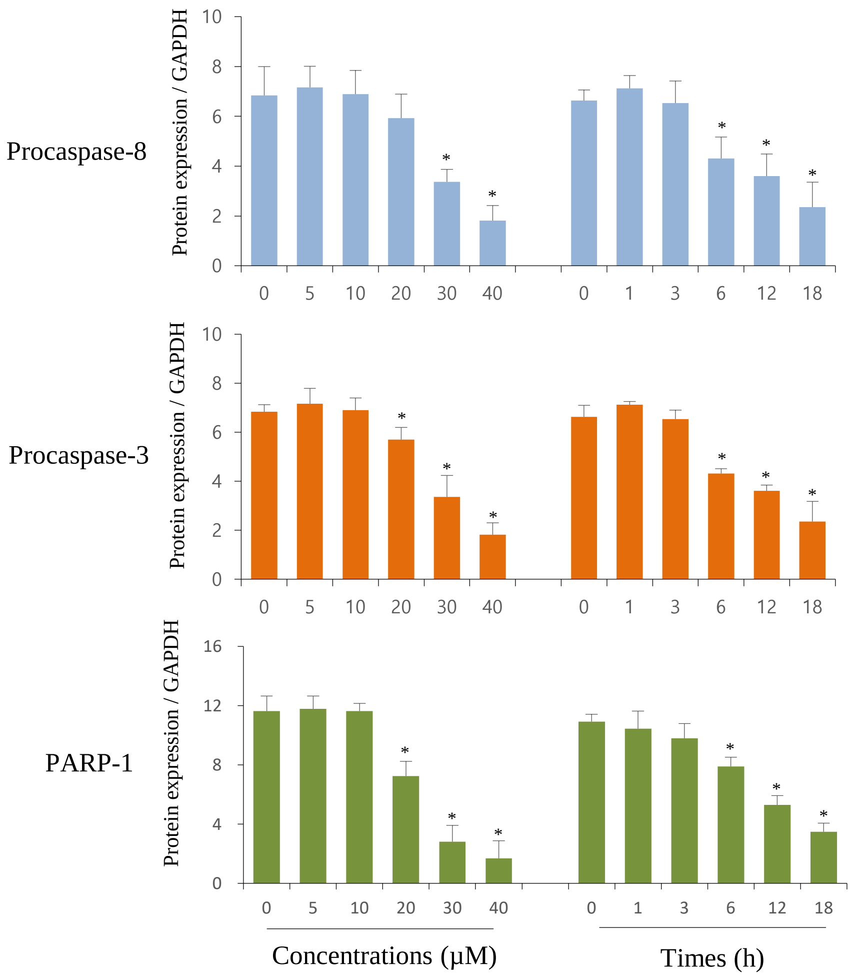

Protein quantification from Fig.1B was measured in optical density units using Image J program (National Institutes of Health, Bethesda, USA) and normalised to the corresponding sample expression of β-actin. n=3, Data were analyzed by *t*-student*t*-test. A value of $^{*}p < 0.05$ versus control was considered statistically significant.

Supplementary Fig. S2 (Oh)

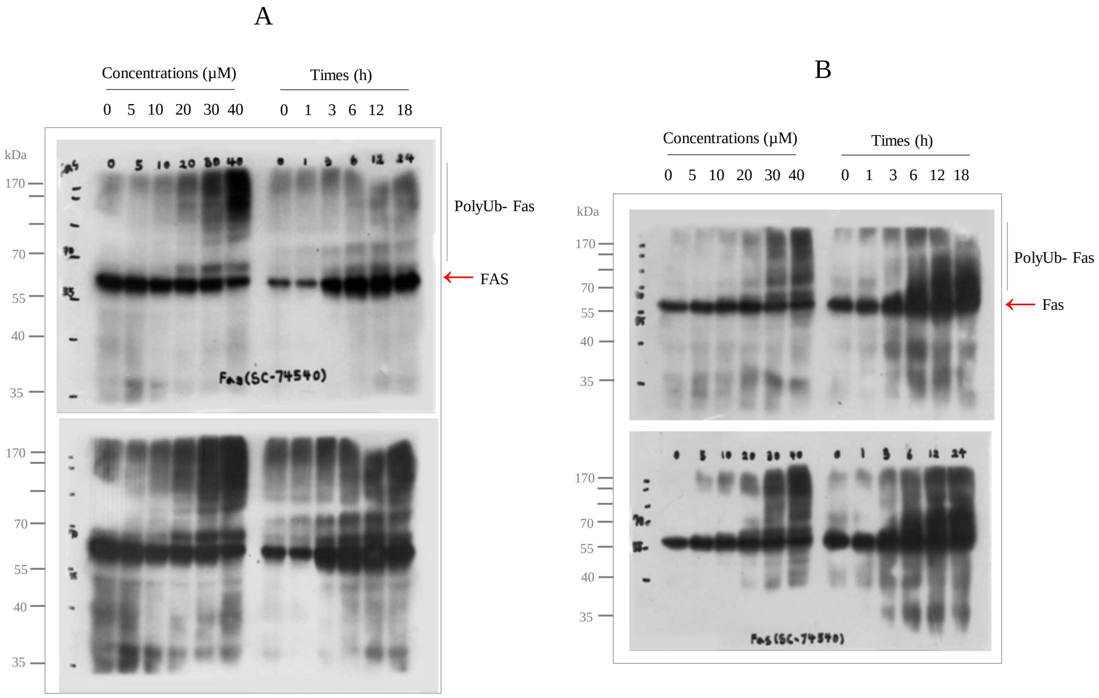

Full blots for full-length Fas (clone G-9). Cells were treated with an increasing concentrations of Cd for 18 h, and with 30 μM for up to 24 h, harvested, lysed, and proteins were separated by SDS-PAGE, analyzed by western blot and probed for full-length Fas (clone G-9) antibody.

Supplementary Fig. S3 (Oh)

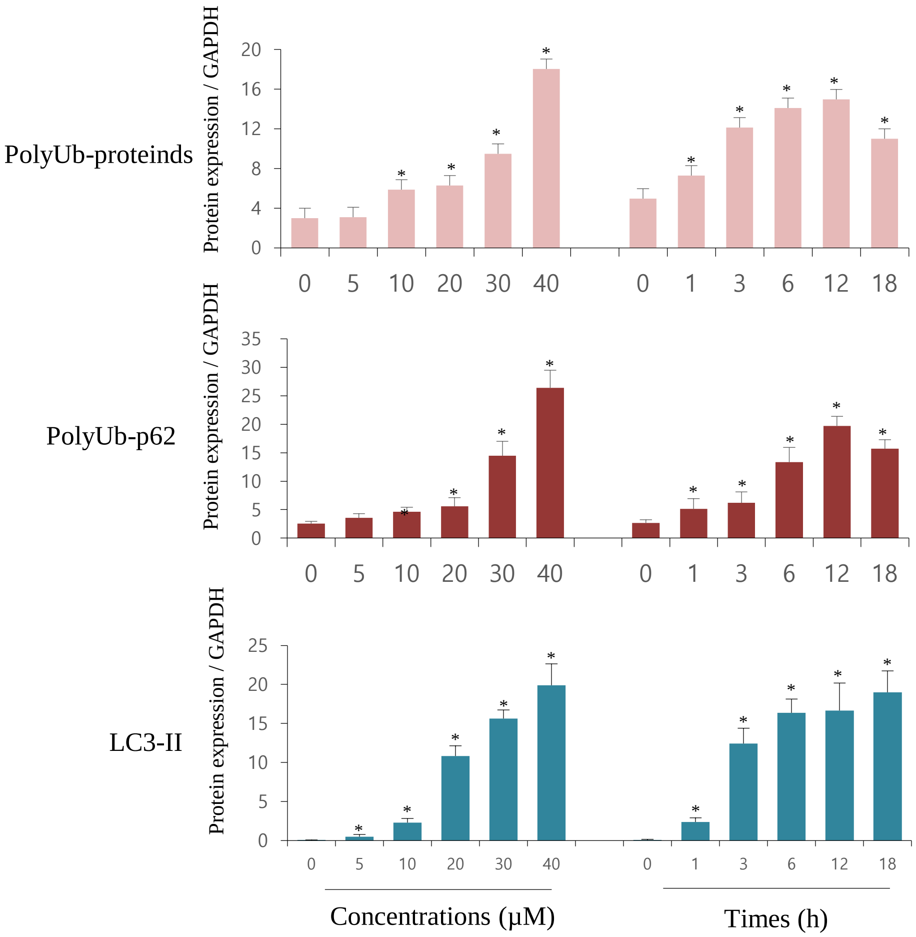

Protein quantification from Fig.2A was measured in optical density units using Image J program (National Institutes of Health, Bethesda, USA) and normalised to the corresponding sample expression of β-actin. n=3, Data were analyzed by *t*-student*t*-test. A value of $^{*}p < 0.05$ versus control was considered statistically significant.

.

Supplementary Fig. S4 (Oh)

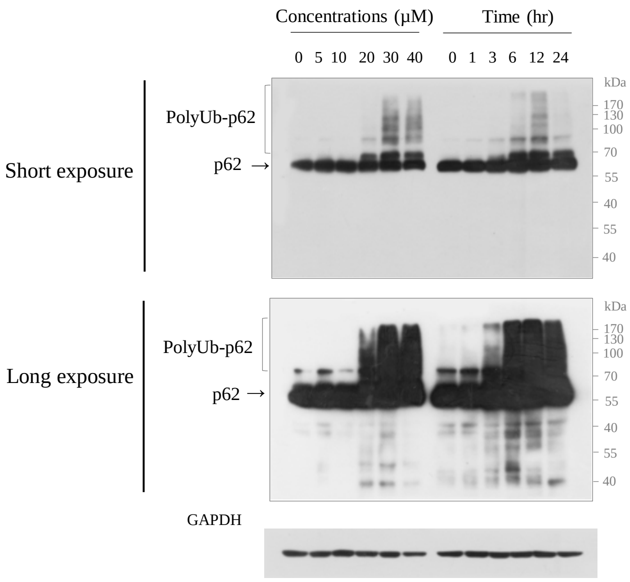

Supplementary Fig. S5 (Oh)

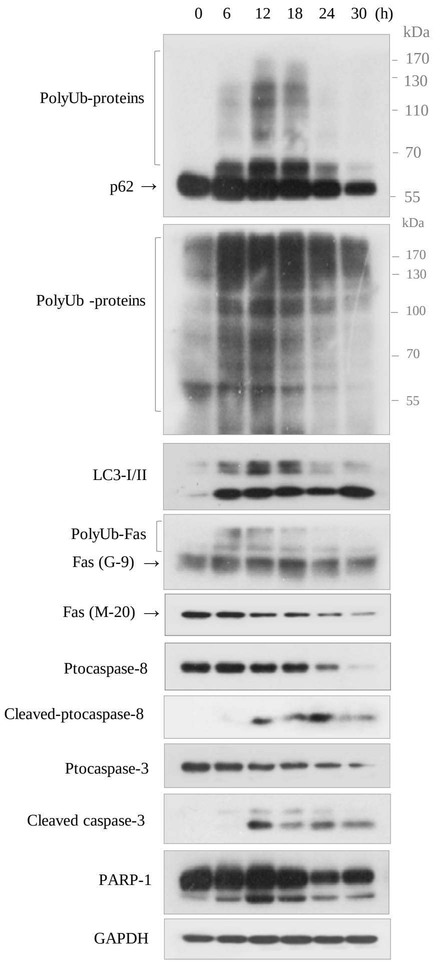

Raw264.7 cells were treated with an IC_{50} concentrations (30 μM) for up to 30 h, harvested, lysed, and expressions of indicated proteins were assessed by immunoblotting. GAPDH was used as the loading control.

Supplementary Fig. S6 (Oh)

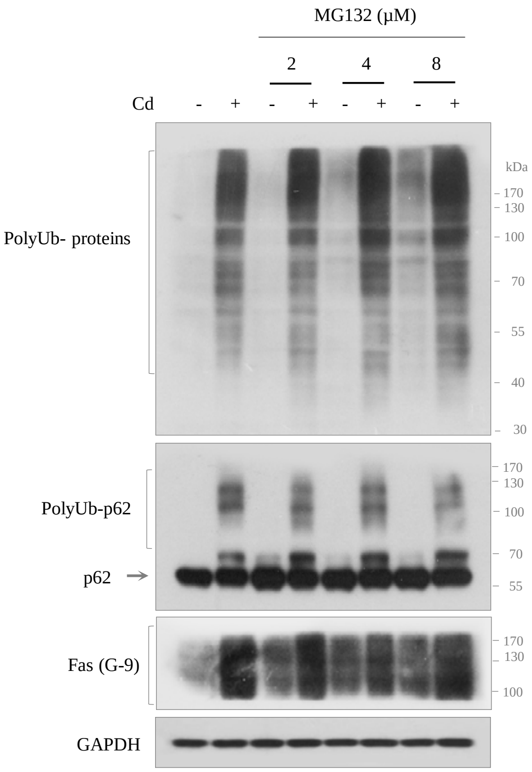

Raw264.7 cells were pretreated with increasing concentrations of MG132 (2~8 μM) and for 2 h and followed by Cd (30 μM) treatment for 12 h, and immunoblotted for indicated proteins. GAPDH was used as the loading control.

Supplementary Fig. S7A (Oh)

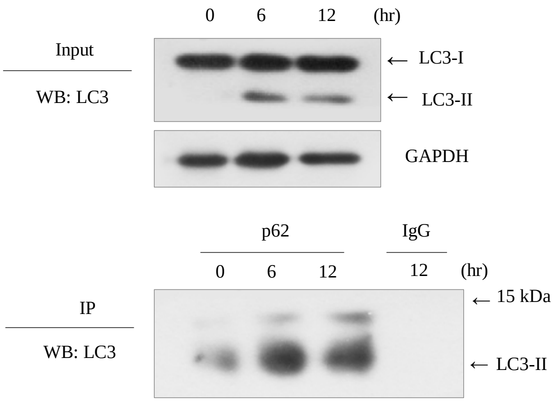

Supplementary Fig. S6B (Oh)

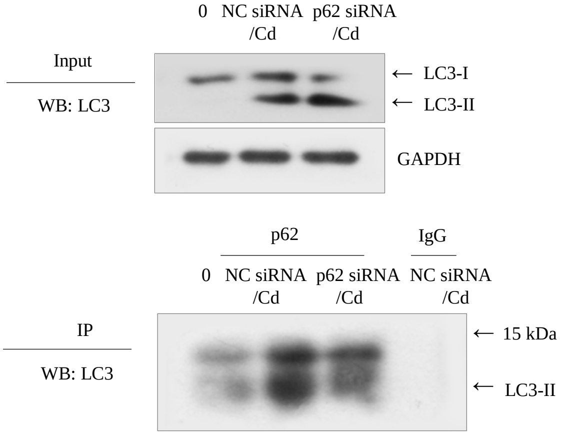

Raw264.7 cells were treated with Cd (30 μM) for 6 and 12 h, respectively. Lysates were immunoblotted for LC3B and the other aliquots (600 μg) were immunoprecipitated with p62 antibody and normal mouse IgG, respectively and immunoblotted for LC3B. GAPDH was used as the loading control. (B) Cells transfected with NC siRNA and p62 siRNA were exposed to Cd (30 μM) for 12 h, and immunoblotted for LC3B and the other aliquots (600 μg) were immunoprecipitated with p62 antibody and normal mouse IgG (NC siRNA/Cd cells), respectively and immunoblotted for LC3. GAPDH was used as the loading control. NC, non-specific control.

Supplementary Fig. S8 (Oh)

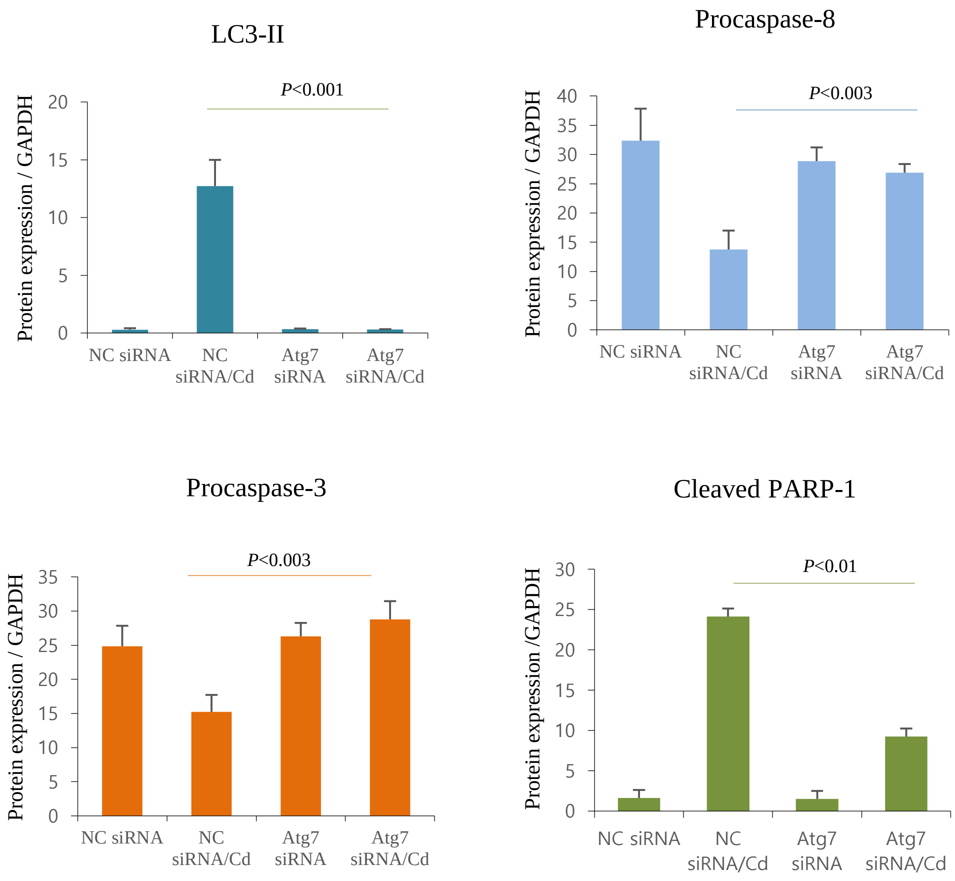

Protein quantification from Fig. 4I was measured in optical density units using Image J program (National Institutes of Health, Bethesda, USA) and normalised to the corresponding sample expression of β-actin. n=3, Data were analyzed by *t*-studen*t*-test.

Supplementary Fig. S9 (Oh)

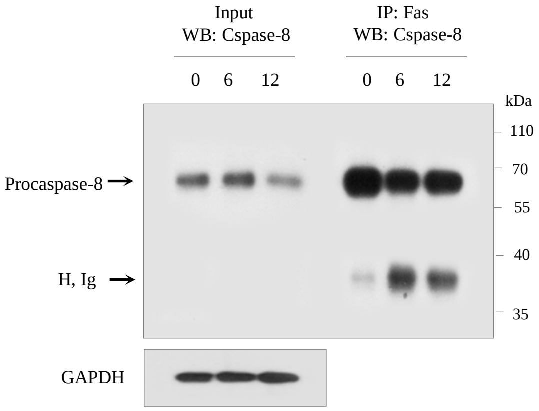

Raw264.7 cells were treated with 30 μM Cd for 6 and 12 h, respectively. Lysates (25 μg) were immunoblotted for caspase-8, and the other aliquot (600 μg) were immuniprecipitated with Fas antibody (clone G-9), followed by immunoblotting for caspase-8. GAPDH was used as the loading control. Data shown are presentative of three separate experiments. H, Ig indicates immunoglobulin heavy chain.
